# Advances in Robotic-Assisted Radical Prostatectomy over Time

**DOI:** 10.1155/2013/902686

**Published:** 2013-11-12

**Authors:** Emma F. P. Jacobs, Ronald Boris, Timothy A. Masterson

**Affiliations:** Department of Urology, Indiana University Medical Center, 535 N. Barnhill Drive, Suite 420, Indianapolis, IN 46202, USA

## Abstract

Since the introduction of robot-assisted radical prostatectomy (RALP), robotics has become increasingly more commonplace in the armamentarium of the urologic surgeon. Robotic utilization has exploded across surgical disciplines well beyond the fields of urology and prostate surgery. The literature detailing technical steps, comparison of large surgical series, and even robotically focused randomized control trials are available for review. RALP, the first robot-assisted surgical procedure to achieve widespread use, has recently become the primary approach for the surgical management of localized prostate cancer. As a result, surgeons are constantly trying to refine and improve upon current technical aspects of the operation. Recent areas of published modifications include bladder neck anastomosis and reconstruction, bladder drainage, nerve sparing approaches and techniques, and perioperative and postoperative management including penile rehabilitation. In this review, we summarize recent advances in perioperative management and surgical technique for RALP.

## 1. Introduction

 Prostate cancer is the most common visceral malignancy diagnosed in American men. The American Cancer Society estimates 241,740 new diagnoses of prostate cancer [1]. It remains the second most common cause of cancer death in American men [1]. Although controversies remain over ideal diagnostic and treatment strategies for prostate cancer, complete removal of the prostate remains the gold standard in the surgical management of localized disease.

Hugh Hampton Young first described the perineal prostatectomy over 100 years ago in 1905 [2]. Subsequently, the first retropubic radical prostatectomy (RRP) was performed by Millin in 1947 [3]. Anatomic studies in the 1970s and early 1980s led to improved appreciation of periprostatic features (dorsal venous complex, endopelvic fascia, autonomic innervation, and striated sphincter) to decrease morbidity of surgery and improve overall outcomes [4, 5]. More recently, in 1997, Schuessler et al. described the first LRP reporting the feasibility of technique despite its association with long operative times [6]. Since that time, numerous European and US centers continued to improve and refine technical aspects of the laparoscopic approach [7, 8].

Several robotic systems were introduced around the turn of the century. The da Vinci system (Intuitive Surgical Inc, CA, USA) was first introduced in 1999. Following a merger with Computer Motion Inc. (AESOP and ZEUS systems) in 2003, Intuitive Surgical has become the sole producer of robotic surgical devices [9]. After initially embarking into cardiothoracic surgery, the da Vinci robot found popularity within the urological community. From the initial descriptions of RALP in 2000 [10, 11], it has become widely adopted by urologists. By 2008, roughly 80% of RPs in the United States were performed robotically [12]. RALP has continued to evolve rapidly since that time with contributions including procedural step by steps, technical modifications, and outcomes data from various surgeons throughout the literature. In this review, we summarize the recent advances in surgical technique and perioperative management of patients undergoing RALP. An overview of significant contributions can be found in Table 1. Major areas of interest which we will address include urinary continence and the vesicourethral anastomosis, bladder and abdominal drainage, modifications to the procedure to minimize erectile dysfunction, and perioperative considerations such as positioning, incision choice, and thromboembolic prophylaxis.

## 2. Methods

A comprehensive review of the published literature was performed using the PubMed search engine. Search terms included robotic prostatectomy, laparoscopic prostatectomy, robotic complications, and robotic technique. English-language search results were reviewed for relevance and then used appropriately. We focused on articles that have been published in the last 5 years, with some review of older sources for a historic perspective. 

### 2.1. Urinary Continence and the Urethrovesical Anastomosis

Urinary continence remains a significant source of morbidity and concern for patients with prostate cancer. Major advances are detailed in Tables 2 and 3. Quality of life questionnaires have demonstrated that urinary control postoperatively may have the greatest impact on a patient's perception of his recovery [13, 14]. As a result, a number of surgical modifications in technique have been used in an attempt to improve early return and overall continence following surgery. Despite numerous published outcomes supporting outstanding recovery of continence following surgery, lack of standardization has led to some controversy. Definitions of continence have ranged from 0 to 1 pad use, 0 pads including a “security pad”, 0 pads, and “leak free, pad free” (LFPF). Additionally, patient-recorded outcomes via questionnaire may significantly differ from surgeon perception. Standardization of RALP outcome definitions (such as continence) is imperative before adequate comparisons of these variables can be made.

A variety of surgical techniques have been employed in an attempt to improve early return of continence after RP (both open and minimally invasive), including bladder neck (BN) preservation [15, 16], intussusception of the BN [17], puboprostatic ligament sparing, sling construction [18], incorporation of the striate urethral sphincter to the anastomosis, and tubularization of the bladder neck. Historically, the actual benefits of these modifications have been somewhat controversial. Many of the surgical reconstruction techniques for RALP have been based on the posterior reconstruction described by Rocco and colleagues in 2001 [19]. In posterior reconstruction, the posterior rhabdosphincter is joined to the posterior Denonvilliers' fascia and fixed to the bladder wall 1-2 cm cranial to the new bladder neck to avoid caudal retraction of the urethrosphincteric complex, prior to completing the standard vesicourethral anastomosis. Additional modifications have been described as posterior (PR), anterior (AR), or complete reconstruction and have been employed with subtle variances by numerous open and minimally invasive prostate surgeons.

The anterior fixation stitch or urethropexy can be performed either prior to or after the anastomosis. As documented by Campenni et al., some patients who have had an anterior urethropexy stitch placed appear to increase continence as measured by leak-point pressures [20]. This maneuver has been described to increase overall continence and decrease time to return of continence [21]. Although many published reconstruction modifications have suggested improved time to continence for RALP, the vast majority have been observational studies with low levels of evidence. By contrast, Menon et al. used a randomized controlled trial to demonstrate no improvement in continence combining an AR and PR (double layered anastomosis) with his standard anastomosis [22]. There were 57 patients randomized to the single and 59 to the double layer anastomotic groups. The study was powered to detect a 30% difference in urinary continence measured in pad weight at various time intervals following surgery. No significant difference between groups was noted. A followup study was performed at two years confirming the author's earlier findings showing excellent urinary control for patients with or without the additional reconstruction [23]. Patients without the double layered technique did demonstrate a higher anastomotic leak rate, but this was not clinically significant. Similar negative results were reproduced in smaller series by Sutherland et al. and Joshi et al. evaluating 94 and 107 patients, respectively [24, 25].

Barbed suture has been theorized to simplify and decrease time for the BN anastomosis. A randomized clinical trial was recently conducted at the Vattikuti Institute in Detroit with 64 patients [26]. Posterior reconstruction was done using a single barbed, 3–0 polyglyconate suture. The urethrovesical anastomosis (UVA) and PR were completed separately using double loaded barbed suture. Knots were not required due to the gripping properties of the suture. The control arm used similar technique with the exception of tying the suture and using an assistant to provide tension on the running anastomosis. The anastomosis was completed more efficiently in the barbed suture arm (4.7 minutes time difference overall). They found no difference in leaks, bladder neck contractures, patient symptoms, or other outcome measures. Kaushik et al. examined the effect of robotic manipulation on unidirectional barbed suture and did find minor structural damage to the barbs and suture on electron microscopy. This was not felt to be clinically significant [27].

Additional published modifications to RALP technique for the improvement of continence include a “pubovesical complex sparing technique” and the use of intraoperative hypothermia. In a small sample of patients, Asimakopoulos et al. described their anatomic dissection plane ventrally between the detrusor apron and the prostate to spare the plexus of Santorini, essentially leaving the dorsal vein complex intact [28]. Using an intraoperative cooling balloon in 109 patients, hypothermia was introduced by Finley et al. to potentially better recover both continence and potency after surgery in a prospective study with a historic control group [29]. The median temperature achieved was 25.5°C. Long-term followup showed that the time to 0 pad status at 3 and 12 months was 81% to 89% and 100% for the initial and extended cooling groups compared with 69% and 89% in the control group, respectively. Return to continence was also faster in the cooling group (39 versus 62 days to zero pads). Although there was some debate as to the ideal temperature goal, duration of cooling, and reproducibility of technique, patients tolerated hypothermia without side effects and early results were encouraging. It is unclear if the authors are continuing to use hypothermia routinely with RALP to date.

Traditionally with RRP, the bladder has been drained via urethral catheter for a finite period of time to maintain urethral patency. Historically, after RRP, catheters were left in place for 2-3 weeks [4]. Despite this precaution, BN contractures were reported in 5–32% of patients [30]. With the running anastomosis and the improved visualization of mucosa to mucosa suturing, postoperative bladder neck contractures have been significantly reduced with RALP to 0–3% [30]. The desire by both patient and surgeon for early catheter removal, or perhaps to obviate the need for urethral stenting altogether, has led to novel methods of bladder drainage. The group at the Vattikuti Urology Institute in Detroit determined that the indwelling urethral catheter was the least tolerated aspect for patients after RALP [31]. This led to the introduction of a 14F suprapubic tube (SP) placed under direct vision following the anastomosis. Patients began cycling their bladder by day 5. The SP was removed when PVRs were less than 30 mL, typically on day 7. Cystography was not used routinely [32]. Continence and stricture rates were similar between this group and historical controls [31]. Another study looked at a custom SP with a small bladder neck anastomotic splint which could be retracted. This pilot study found earlier return of continence and decreased patient discomfort [33]. Longer followup with larger cohorts will be needed to evaluate the possible downstream effects of the total removal of urethral catheterization (continence rates, leak rates, and bladder neck contracture rates) following RALP, but early results are intriguing.

In summary, the overwhelming majority of patients will eventually recover continence after RALP. Despite this notion, time to continence and achievement of a true “pad free, leak free” state may be impacted by numerous factors and frequently subject to poor reporting, patient and physician biases, and limited utilization of accepted quality of life questionnaires. As the vast majority of patients undergoing RP will ultimately succumb to diseases other than prostate cancer, recovery of functional outcomes after surgery may have more impact on the “perceived success” of surgery compared to surgery for any other surgical malignancy. Hence, we continue to strive to find new ways to deliver continence quicker and better despite an acceptable status quo demonstrated in most post-RP health related quality of life studies [34]. Whether or not these described technical modifications to RALP truly improve recovery of continence, they appear safe, are relatively easy to perform, and carry little risk.

### 2.2. Erectile Dysfunction

Several surgical modifications in the technical approach to RALP have resulted in vast improvements in understanding the neuropathic basis behind the development of erectile dysfunction. Prominent studies are detailed in Table 4. Historical rates of erectile dysfunction after RRP were near universal until the neuroanatomy of the pelvic plexus responsible for physiological erections was described by Walsh and Donker [35]. In the subsequent era of the anatomic RRP, erectile dysfunction rates improved dramatically. Clinical features that predict better outcomes for sexual function recovery include patient age, quality of erections prior to surgery, clinical stage, and the quality and extent of neurovascular bundle preservation [36–38]. While reported rates vary, centers of excellence achieve 12-month potency rates with or without the use of phosphodiesterase-5 inhibitors ranging from 68% up to 91% with bilateral nerve sparing using RRP techniques [36, 37, 39, 40]. While disparate data exists with outcomes for sexual function after RALP, high volume centers have published comparable outcomes ranging from 70% to 80% at one year [41–43].

Several centers, including the group at Henry Ford Hospital in Detroit, Michigan, have offered their experience in surgical technique leading to the evolution of RALP. The VIP approach to RALP was first described in 2002 [44], followed by several refinements leading to the current description [41]. Among these refinements are the development and preservation of the lateral prostatic fascia (i.e., veil of Aphrodite). This involves releasing the lattice or curtain of cavernosal nerve tissue extending along the posterolateral aspects of the prostate bilaterally, up to the fibrous stroma of the dorsal vein complex anteriorly overlying the apex of the prostate [41]. In a more recent modification, a “superveil” technique was developed for select patients with favorable anatomy allowing for further extension anteriorly of the dissection to preserve the pubovesical ligaments and dorsal vein complex [45]. In both circumstances, erectile function recovery rates in the most favorable patient groups were 93% and 94%, respectively. 

The rationale for these modifications was based upon cadaveric dissections suggesting that smaller nerves of the pelvic neurovascular plexus exist along the prostatic and Denonvilliers' fascia [46]. Further cadaveric studies have supported the concept of a neural “hammock” extending from the posterior surface of the seminal vesicles down along the posterolateral aspects of the mid-prostate, and diverging anterolaterally near the membranous urethra [47–49]. Based upon these studies, a “trizonal” neural hammock concept including the proximal neurovascular plate posterior to the seminal vesicles, the predominant neurovascular bundle along the posterolateral aspect of the prostate, and the accessory neural pathway was characterized [50]. Using similar concepts, Srivastava and colleagues recently published a review of their current technique for neurovascular bundle (NVB) preservation [51]. Building upon basic tenants of the RRP, their approach includes avoiding unnecessary traction and thermal injury to the neurovascular bundle throughout the trizonal region. Through a medial to lateral approach beginning with the seminal vesicles and extending along the posterolateral aspect of the prostate, the authors used sharp, athermal dissection and small pedicle clipping to facilitate intrafascial dissection. Once released posterolaterally, further dissection allows easy mobilization of the anterolateral lattice of accessory nerves from the base towards the membranous urethra. In their retrospective analysis of 2317 consecutive patients undergoing RALP, 91% of men with the most favorable characteristics undergoing these technical modifications reported the ability to engage in sexual intercourse [52]. For men aged 60 years or less, rates of erectile function recovery beyond 12 months were 95% with grade 1 NVB preservation. These rates incrementally deteriorated with progressively wider planes of dissection resulting in greater degrees of NVB resection (i.e., grades 2–4).

The impact of countertraction on NVB injury and secondary neuropraxia has been further emphasized in a recent report by Kowalczyk and colleagues [53]. In their single surgeon experience among 610 patients undergoing RALP, 342 of the procedures were performed while avoiding assistant and/or surgeon countertraction of the NVB during nerve sparing RP. This was accomplished by placing traction on the prostate itself and dissecting it away from the NVB. These patients experienced earlier time sexual function recovery (45% versus 28% at 5 months), but overall potency rates at one year were similar between groups. This is in line with similar results reported with RRP in techniques developed to avoid traction on the NVB [54]. The investigators from Bordeaux, France, reported on their lateral technique to NVB preservation [55]. In their approach, the NVB is released prior to division of the bladder neck fibers and seminal vesicle dissection to facilitate a tension-free release to the NVB preservation. While no comparison cohort is offered, they report a high rate of spontaneous erections (not necessarily sufficient for intercourse) in 46% of patients within one week after catheter removal. Additionally, 65% of patients were considered potent at the 4-month followup visit. No results at the 12-month time period were reported for comparison. 

Regarding the effect of thermal energy on the recovery of potency after RALP, limited clinical data exists to draw significant conclusions. While monopolar cautery is generally accepted to be associated with significant thermal injury to nervous tissue when applied, equipoise remains as to the impact of bipolar cautery during NVB preservation. In a study utilizing thermal probes intraoperatively during RALP, Mandhani et al. demonstrate a substantial increase in regional temperature of the NVB when bipolar cautery was used compared to monopolar current [56]. While differences in its impact on sexual function have not been reported, the author raises the question as to whether bipolar current is truly safer. Questions as to the importance of thermal injury were also raised by Khan et al. Using fiber optic thermometry, they found that when bipolar and monopolar currents were applied to the anterior abdominal wall, the inferior epigastrics were able to act as a heat sink. This was extrapolated to suggest that the prostatic pedicle should be protective of the neurovascular bundle during bladder neck dissection [57]. Nevertheless, it is easy to suspect that thermal injury may contribute to loss of potency at some level. Subsequently, several investigators have attempted to determine the feasibility of performing completely athermal RALP. Ahlering et al. reported that utilizing bulldog clamps on the hypogastric vessels to potentially eliminate the need for cautery during prostate dissection was feasible and resulted in a nearly 5-fold rate of improvement in sexual function recovery [58]. Similarly, Chien et al. from Chicago reported similar findings during a completely athermal RALP, stating that their results led to a quicker return and preservation of potency [59]. Most recently, Gill et al. demonstrated using real-time Doppler transrectal ultrasound that bulldog clamping of the lateral vascular pedicle preserved NVB blood flow while minimizing cautery need [60]. 

Overall, general principles of NVB preservation remain steadfast. Prospective anatomic identification of location and routes of primary and accessory neurovascular bundles combined with the minimization of countertraction, tension, and thermal injury to these bundles will optimize the technical success of the operation in preserving and recovering sexual function in well-selected patients. 

## 3. Perioperative Considerations 

As with all major operations, surgeons constantly strive to minimize perioperative complications and their associated morbidity from the time the patient enters the operating room until their recovery is complete. In some circumstances, prevention of complications can begin prior to surgery. Because RALP is an overall well-tolerated procedure for a relatively healthy patient population for a malignancy associated with high survival rates, perioperative morbidity is highly scrutinized. Potentially devastating consequences may occur from subtle nuances associated with the use (or absence) of venous thromboembolism prophylaxis, patient padding and positioning, operative times, and trocar placement and/or incision choice. 

The incidence of venous thromboembolism (VTE) associated with robotic or laparoscopic prostatectomy is roughly 0.5% [61]. Additionally, the actual incidence is likely much higher as VTE is typically only reported when it is symptomatic. The Best Practice Statement on deep venous thrombosis recommends intermittent pneumatic compression (IPC) for laparoscopic and robotic procedures, with the possible addition of chemoprophylaxis in high risk groups [62]. A large multi-institutional retrospective review concluded that VTE prophylaxis is not indicated in RALP (without additional risk factors) [61]. Preoperative prophylaxis may lead to significantly increased blood loss [61]; however, the increased morbidity and mortality associated with VTE certainly makes it an issue of concern. The mortality of RRP overall is 0.1%, which is increased to 6% in patients with VTE. Risk factors include malignancy, immobility in the perioperative period, and medical comorbidities. A recent study by Schmitges et al. found that the surgeon's overall annual caseload also had an effect on the rate of DVT and PE [63]. They found a rate of 0.1% with surgeons doing more than 24 RALPs yearly, 0.3% with 10–24 cases, and 0.3% with less than 10 cases. These authors also did not find a difference in the incidence of VTE between open and minimally invasive techniques. As more patients undergo extended lymph node dissection during RALP, it remains unclear what if any impact this will have on the future rates of VTE in this patient population. Without question, future investigation will be needed to better define which patients are best candidates for VTE prophylaxis and at what doses and durations should patients be treated. 

During standard RALP, the patient is typically secured with his legs apart in the split-leg position or in dorsal lithotomy, followed by transitioning into a steep Trendelenburg position (25–35 degrees). This allows for positioning of the robot system, as well as providing access for the assistant to manipulate the catheter during the anastomosis [64]. Proper padding, positioning, and assuring that the robot remains a safe distance from the patient are key components to avoid compartment syndromes and other compressive injuries, which can ultimately lead to myonecrosis and renal insult. The hallmark of compartment syndromes on physical exam is pain on passive stretch. The rate of lower extremity neuropathies in split-leg position is roughly 1.3%, with increased intraoperative time being the only factor studied which increased risk [65]. This position increases the risk of injury to the femoral nerve [65]. At 3 hours, significant ischemia-reperfusion injury occurs [66]. Modified lithotomy, heel support, and avoidance of the Trendelenburg position and ankle dorsiflexion, as well as the use of intermittent compression devices (many of these features not feasible with RALP), have all been found to reduce the risk of intraoperative compartment syndromes [67]. Although rare, case reports of gluteal compartment syndromes have been associated with dorsal lithotomy positioning and may occur bilaterally with untoward results [68]. Statistics on the incidence of myonecrosis after RALP are not available in the literature, although it can be presumed to be a rare and largely preventable complication. 

The need for placement of an abdominal drain for the potential collection of urinary extravasation prior to closure has become somewhat controversial in RALP. Despite being relatively well tolerated, most drains play little role in the postoperative course of men undergoing RALP. Drains are commonly removed within 24 hours prior to discharge whenever placed and are many times already out in the unusual circumstance when a patient re-presents with symptoms of urinary ascites. Sharma et al.'s 2007 review of 225 open and 100 robotic cases did not find that drainage improved recovery or complication rates in either group [69]. Selective drain placement when the integrity of the anastomosis is in doubt was supported by another review from Lahey Clinic [70]. The overall trend supports moving away from routine abdominal drainage.

The rate of incisional hernias after RALP is poorly defined, largely because it is not followed as a long-term endpoint. Most hernias develop at the periumbilical incision, although more rarely the smaller port sites have developed hernias as well [71]. Port site hernias may have significant morbidity as the smaller size of the defect can lead to entrapment and strangulation of bowel and omentum. The risk of a trocar site hernia is 0.8–1.2% [72]. The rate of hernia occurrence is higher with 12 mm trocars (as opposed to 10 mm or 5 mm) [73] and with cutting trocars (as opposed to blunt or radially expanding) [74]. However, overall hernia incidence has been estimated at 3.3–16.7%, with great variation found between different methods of followup [75]. Factors that can contribute to hernia development include patient comorbidities, technical issues with wound closure, and the choice of incision. Previous review in the surgical literature [76] has suggested that a transverse incision has a statistically significant advantage in terms of hernia occurrence. Beck et al. [75] found that a transverse incision in RALP can reduce the rate of hernias. Fuller et al. determined that closure of the midline incision with nonabsorbable suture using interrupted stitches can decrease hernia occurrence [71]. Early recognition is a key in preventing severe complications secondary to postoperative hernias. Instrument and suture selection, as well as careful wound closure, can avoid these complications. 

Inguinal hernias have been reported as a complication of RRP, originally reported as appearing in 12% of postprostatectomy patients as opposed to 5% in the male population [77]. Rabbani et al. concluded that risk factors included advanced age, low BMI, prior inguinal hernia repair, and bladder neck contracture [78]. This has been theorized to be due to the lower midline incision and subsequent weakening of the abdominal wall [79]. Nerve sparing prostatectomies (open, laparoscopic, and robotic) have also been associated with higher rates of inguinal hernias requiring operative repair [80]. 

Perioperative complications can often be reduced by awareness and attention to details. Careful positioning, reduced operative time, selection of operative equipment and suture, and prophylaxis in some circumstances can avoid preventable complications. 

## 4. Robotic Technologic Advances

Continued advances in both modern medicine and technology constantly drive change of current technique. As we continue to emphasize a shift toward “minimally invasive surgery,” industry has developed new concepts employing fewer ports and tinier incisions while trying to maintain similar outcomes. Laparoendoscopic single site surgery (LESS) has yet to become widespread in urology but is gaining ground in the robotics arena. Currently, single-site robotic instruments and techniques have been better described for upper tract and pediatric indications [81] than pelvic and/urologic surgery. White et al. concluded after a retrospective review of 50 robotic LESS procedures that a redesigned or task specific robotic platform would be required before this technique could be adopted for widespread use [82]. LESS RALP was described in 2010 using multiple ports in a single umbilical incision. Of 20 patients undergoing this procedure, 2 required additional ports and 1 was converted to a traditional RALP [83]. Challenges with LESS surgery include adjusting to the necessary curved instruments and the limited range of motion. Florescent-sensitive cameras are a recent innovation which may provide functional intraoperative imaging. Protoporphyrin IX (PpIX) precursors delta-aminolevulinic acid (*δ*ALA) and hexyl aminolevulinate (HAL), fluorescein, and indocyanine green (ICG) have been approved by the FDA for clinical use. ALA has been used experimentally to visualize prostate tissue [84]. Newer daVinci equipment is florescence capable. The clinical applications of this have not been fully established.

## 5. Conclusions

Over the past ten years, both domestically and worldwide, there has been explosive growth in the use of robotic-assisted laparoscopic prostatectomy as an alternative to open radical retropubic prostatectomy. Some advantages of the minimally invasive approach have been well established, while others, particularly those associated with functional outcomes, remain controversial. Multiple factors, including the drive to improve oncologic outcome, patient comfort, and quality of life, have driven refinement in technique as well as perceived “success” of the operation. Our review outlines the evolution of robotic prostatectomy through both improvements in functional outcomes as well as the minimization of patient discomfort and overall morbidity. Whether or not these described technical modifications to RALP truly improve upon these elements remains unknown. Surgeons should be encouraged to evaluate their own results and introduce any one or more of these changes if their results are below standard of care.

## Figures and Tables

**Table 1:** Modifications to RALP.

	Modifications	Year introduced	References
Urinary continence	Sling construction	1997	Jorion [18]
	Bladder neck preservation	2002	Deliveliotis et al. [16], Selli et al. [15], and von Bodman et al. [85]
	Intraoperative cooling	2009	Finley et al. [29]
	Pubovesical complex sparing	2011	Asimakopoulos et al. [28]

UVA	Posterior reconstruction	2008	Rocco et al. [19, 86], Coelho et al. [87]
	Anterior reconstruction	2009	Campenni et al. [20], Patel et al. [21]
	Double layer anastomosis	2009	Menon et al. [22], Sammon et al. [23], and Joshi et al. [25], Sutherland et al. [24], Sammon et al. [23], and Hurtes et al. [88]
	Barbed suture	2011	Sammon et al. [26], and Kaushik et al. [27]

NVB sparing	NVB sparing	1991	Quinlan et al. [36], Catalona et al. [39], Dubbelman et al. [38], Rabbani et al. [37], and Walsh et al. [40]
	Veil of Aphrodite	2002	Menon et al. [41, 45]
	Athermal dissection	2007	Tewari et al. [52], Mandhani et al. [56], and Khan et al. [57], Ahlering et al. [58], Chien et al. [59], and Gill et al. [60]
	Tension-free	2007	Kowalczyk et al. [53], Mattei et al. [55]

Bladder drainage	SP drainage	2009	Krane et al. [31], Sammon et al. [32], and Tewari et al. [33]

**Table 2:** Urinary continence.

Modification	Primary author	Results
Sling construction	Jorion [18]	Fascial sling suspension after anastomosis in RRP resulted in earlier and more complete continence.

Bladder neck preservation	Deliveliotis [16]	Return of continence was earlier after RRP in bladder neck preservation group compared to puboprostatic ligament sparing or both techniques used together. Final continence rates were unchanged.
	Selli [15]	Bladder neck preservation in RRP leads to faster return of continence but does not affect long-term recovery.
	von Bodman [85]	Anatomic variables membranous urethral length, urethral volume, and an anatomically close relation between the levator muscle and membranous urethra on preoperative magnetic resonance imaging are independent predictors of continence recovery after radical prostatectomy.

Intraoperative cooling	Finley [29]	Regional pelvic cooling during RRP was associated with early return of continence. Longer and deeper cooling improved continence.

Pubovesical complex-sparing	Asimakopoulos [28]	128 patients were randomized to LRP or RALP. Erectile function at 12 months was better in the RALP group. Oncologic outcomes and continence were similar between the two groups.

**Table 3:** Urethrovesical anastomosis.

Modification	Primary author	Results
Posterior reconstruction	Rocco [19, 86]	Posterior reconstruction in RRP was associated with improved time to return to continence.
	Coelho [87]	Posterior reconstruction in RALP had faster return of continence and fewer anastomotic leaks.

Anterior reconstruction	Campenni [20]	Anterior anastomotic urethral suspension sutures increase Valsalva LLP and may speed the return of continence.
	Patel [21]	Suspension stitch in RALP leads to improved continence at 3 months.

Double layer anastomosis	Menon [22]	No improvement in continence was seen with reconstruction.
	Sammon [23]	Single or double layer anastomosis did not correlate to urinary outcomes at 2 years.
	Joshi [25]	Posterior reconstruction did not improve continence after RALP.
	Sutherland [24]	Posterior reconstruction did not increase early return of continence after RALP.
	Hurtes [88]	Early return of continence after RALP was improved with anterior suspension combined with posterior reconstruction.

Barbed suture	Sammon [26]	RALP using v-lock suture showed a decrease in anastomotic time without change in outcomes.

**Table 4:** Neurovascular bundle sparing.

Veil of Aphrodite	Menon et al. [41]	VIP with veil nerve sparing has comparable outcomes to traditional RALP.
	Menon et al. [45]	VIP had improved erectile function compared to RALP.

Athermal dissection	Tewari et al. [52]	Neural hammock sparing improves return to baseline erectile function without affecting other outcomes.
	Khan et al. [57]	Heat sink demonstrated in this porcine model suggests that the vascular pedicle should be protective of the NVB.
	Ahlering et al. [58]	Avoiding thermal injury leads to earlier return of sexual function.
	Chien et al. [59]	Antegrade athermal dissection may lead to earlier return of erectile function.

Tension-free	Kowalczyk et al. [53]	Avoidance of countertraction on the NVB leads to earlier return of erectile function.
	Mattei et al. [55]	This lateral approach to the NVB is tension-free and athermal.

## Abbreviations

AR: Anterior reconstruction
CR: Complete reconstruction
BN: Bladder neck
ED: Erectile dysfunction
LESS: Laparoendoscopic single site surgery
LFLP: Leak free, pad free
NVB: Neurovascular bundle
MUL: Membranous urethral length
PR: Posterior reconstruction
RALP: Robot-assisted laparoscopic (radical) prostatectomy
LRP: Laparoscopic radical prostatectomy
RP: Radical prostatectomy
RRP: Radical retropubic prostatectomy
SHIM: Sexual Health Inventory for Men
SP: Suprapubic
VIP: Vattikuti Institute Prostatectomy
VTE: Venous thromboembolism
UVA: Urethrovesical anastomosis.
